# Synergy between infection control and antimicrobial stewardship programs to control carbapenem-resistant Enterobacterales

**DOI:** 10.1017/ash.2023.439

**Published:** 2023-09-26

**Authors:** Wanda Cornistein, Daniela Santonato, Paula Andrea Novau, Leonardo Guillermo Fabbro, Melisa Fernanda Jorge, Maria Agustina Malvicini, Viviana Vilches, Fernando Martin Iudica

**Affiliations:** 1 Infection Prevention and Control Department, Hospital Universitario Austral, Buenos Aires, Argentina; 2 Department of Phamacy, Hospital Universitario Austral, Buenos Aires, Argentina; 3 Microbiology Laboratory, Hospital Universitario Austral, Buenos Aires, Argentina; 4 Medical Director, Hospital Universitario Austral, Buenos Aires, Argentina

## Abstract

**Objective:**

Argentina is the third country in the world with the higher levels of CRE. The primary objective is to achieve an optimal result in the CRE infection rate after the implementation of an IPC program and antimicrobial stewardship programs (ASP) in a large teaching hospital in Argentina.

**Methods:**

Retrospective, observational study from January 2018 to December 2021, in a 220-bed tertiary care teaching hospital in Buenos Aires province. Actions aimed at CRE control and prevention included CRE and healthcare-associated infection (HAI) surveillance; compliance with hand hygiene, hospital hygiene, contact isolation precautions, and care bundles for the prevention of device-associated infections; optimization of antimicrobial treatments, antimicrobial consumption, education, and feedback.

**Results:**

Synergy between an ICP and ASP achieved controlled rate of CRE infections reaching the lowest levels during 2020 (0.08 episodes/1000 patient days). Colonization rate remained stable throughout the study period. Ventilator-associated pneumonia (VAP) rate showed a trend toward lower rates. Compliance with care bundles showed rates >85%. Antimicrobial consumption increased slightly during the study period (15%). Among high-impact antimicrobials, only colistin consumption increased.

**Conclusion:**

Our study demonstrates the sustained and beneficial impact of an IPC Program and an ASP to control CRE infection.

## Introduction

Antimicrobial resistance (AMR) rate increase constitutes a global threat and is projected to be one of the leading causes of death by 2050^1^ accounting for 10 million deaths each year. Some contributors of AMR include improper and excessive use of antimicrobials in both, humans and animals, coupled with inadequate infection control and prevention measures.^2^

Carbapenem-resistant Enterobacterales (CRE) are a growing problem for public health. Detection and control of CRE infections are a challenging issue, mainly in low- and middle-income countries (LMIC). During COVID-19, countries in Latin America reported clinical emergence of carbapenemase-producing Enterobacterales that had not been previously characterized locally, increased prevalence of carbapenemases that had previously been detected, and co-production of multiple carbapenemases in some isolates.^3^

Argentina is the third country in the world with the higher levels of CRE,^4^ being 43.5% carbapenem-resistant of the whole *K. pneumoniae* isolates in 2020.

A recent study—which included 822 CRE isolates from 183 hospitals—detected at least one carbapenemase gene in 97.3% of the isolates. Main genes were blaNDM (41.6%) and blaKPC (39.4%), and 8.7% of the strains presented a combination of genes.^5^

Most multidrug-resistant organisms (MRO) are linked to healthcare-associated infections (HAI). According to a report from the World Health Organization (WHO), HAI are among most frequent adverse events in health care^6^; it is estimated that 15 out of every 100 patients will experience a HAI in LMIC. The significant burden of MRO infections underscores the importance of implementing measures for their control and prevention.^6^

Most healthcare institutions in Argentina lack Infection Prevention and Control (IPC) programs. Argentina is included in the 54% of countries worldwide with a national IPC program and operational plan and the availability of national guidelines for infection prevention.^6^ Nonetheless, approximately only 0.4% of hospitals in Argentina report their HAIs rates to the National Surveillance Program in 2018.^7,8^

Antimicrobial stewardship programs (ASP) constitute another key tool in the battle against AMR. Their primary objective is to achieve optimal patient outcomes for those with suspected infections, while minimizing the unintended consequences of antimicrobial use.^9^

Most basic components required to establish ASP are not available in Argentina^10,11^: limited formal hospital leadership support, inadequate staffing and tools to perform AS work more efficiently, limited awareness of AS principles by HCWs, and limited training opportunity. This situation halts the establishment and articulation of both programs, with heterogeneous results nationwide.

The main objective is to achieve an optimal result in the CRE infection rate after the implementation of an IPC program and ASP in a large teaching hospital in Argentina.

## Material and methods

Retrospective, observational study from January 2018 to December 2021 in a tertiary care teaching hospital in Buenos Aires province. The institution has been accredited from the Joint Commission International since 2013.

The hospital has 220 beds, with 64 critical care beds distributed as follows: (1) adult intensive care unit (ICU) 21 beds, (2) pediatric ICU 10 beds, (3) coronary unit 15 beds, and (4) neonatal ICU 18 beds.

Beginning in 2018, the ICP staff consists of three infection control nurses and two infectious diseases specialists. The ASP has clinical pharmacist, six infectious diseases specialists (four adults and two pediatrics), one member of each of the following: Department of Quality & Patient Safety and the Microbiology Department. Both programs are coordinated by the head of the Infection Prevention and Control Department, which are supervised by the Medical Director.

Programs were evaluated on annual basis using validated tools (WHO infection prevention and control assessment framework and Antibiotic stewardship program assessment tool CDC).

Afterwards, a risk matrix was used to establish priorities. Actions aimed at CRE control and prevention include the following:

1. HAI surveillance. Active surveillance occurs through daily rounds in the hospital by IPC professionals. A registry of patients placed under isolation precautions and/or with invasive devices is maintained. For the definition of episode of HAI infection, the definition of the national surveillance program VIHDA was used (Appendix 1).
2. Carbapenem-resistant *Enterobacterales (CRE)* surveillance. Upon admission, nurses obtained a rectal swab to screen for CRE in patients transferred from other institutions, those with chronic hemodialysis, or those with a history of infection or colonization by MRO. Screening is repeated weekly in Intensive Care Units (ICUs) and Bone Marrow Transplant Unit (BMT) for patients with previous negative results.
  CRE colonization was divided into two categories: 1. CRE detected <72 h after admission (community or other hospital); 2. CRE identified >72 h after admission (nosocomial). The surveillance included screening by rectal swabs and any clinical sample with CRE during the hospitalization without signs and symptoms of infection. CRE infection was defined as cultures nondoubled in patients with systemic signs of infections.
  Rectal swabs are initially processed using selective and differential chromogenic media (SUPERCARBA^®^). Subsequently, positive samples are processed with a rapid colorimetric biochemical test (Blue-Carba^®^) and immunochromographical tests in order to detect carbapenemase-producing *Enterobacterales*. The identification is thereafter complimented with automated methods to determine antimicrobial susceptibility. Culture results are reviewed daily by professional IPCs to report the presence of CRE in clinical specimens and implement isolation.
3. Hand hygiene compliance monitoring. The institution adheres to the WHO Multimodal Hand Hygiene Improvement Strategy. From 2018 to 2020, it was comprised of direct observations including both the moments and technique proposed by the WHO. From 2021, video observation was added to direct observation in critical care units in order to reduce the Hawthorne effect. Observations were performed throughout one week every three months in all units with at least 50 opportunities per unit. The acceptable standard was set at a lower limit of an 80% compliance rate.
4. Hospital hygiene (HH) monitoring. Compliance was monitored monthly, randomly, while patients were hospitalized and at discharge. The observer marked—using invisible fluorescent ink—frequently touched surfaces of patient’s rooms, and, 24–48 h later, the room was inspected with a special lantern recording the remaining marks. The rate of hospital hygiene effectiveness was determined by the percentage of marks no longer visible. At the beginning, the designated observer was the coordinator of Environmental Health Services and in 2021 IPC professionals replaced him.
5. Compliance with contact isolation precautions. Since 2020, appropriate signage, availability of personal protective equipment, proper hand hygiene, and appropriate donning and doffing of supplies were evaluated by IPC professionals monthly in all the hospital units, including emergency department.
6. Compliance with care bundles for the prevention of device-associated infections. IPC specialists and bedside nurses verified adherence to care bundles for the maintenance of central catheter, urinary catheter, and mechanic ventilation daily. The information was registered using MEG^®^ Audit Tool, a cloud-based digital quality management system that allows for bedside registry of data.
7. Compliance with guidelines was audited by clinical pharmacist every three months and reported to prescribers twice annually. Infectious disease practitioner and clinical pharmacist performed a daily antimicrobial prescription audit of hospitalized patients (prioritizing ICU patients) and gave recommendations—feedback—to prescribing physicians.
*Clostridioides difficile* infection, urinary tract infection, catheter bloodstream infection, community and hospital-acquired pneumonia, skin and soft tissue infections, surgical antibiotic prophylaxis, spontaneous bacterial peritonitis, infections in immunocompromised patients, prosthetic joint infection, and empirical treatment for ICU patients.
8. Antimicrobial consumption. The quantification of antimicrobials vials prescribed in adults per semester started in 2018 with the incorporation of the pharmacist. In 2020, antimicrobial ordered is reviewed every month, stratified by inpatient hospital service and drug.
9. Education. The IPC and ASP group developed an educational slide deck addressing key IPC practices, antibiotic guidelines, and update them annually. All newly hired and existing healthcare workers must review these slides each year. Virtual education started in 2020.
10. Feedback. Information on HAI is presented weekly to critical areas and the Medical Board, monthly at Morbidity and Mortality meetings, and every three months at Infection Control Committee meetings.

Table 1 summarizes the IPC and ASP strategies.

**Table 1.** IPC and ASP programs

	2018	2019	2020	2021	Frequency	Responsible
HAI Surveillance	x	x	x	X	Monthly	IPC
MRO Surveillance*	X	x	x	X	Diary	IPC/microbiology/nurses
Hand Hygiene compliance	Direct observation	Direct observation	Direct observation	Direct observation and cameras in critical units	Quarterly	IPC
Hospital Hygiene compliance by fluorescence	x	x	x	X	Monthly	Environmental Health Service/2021 IPC
Contact Isolation compliance	No	No	x	X	Monthly	IPC
Care bundles compliance	IPC	IPC	IPC and nurses	IPC and Nurses	three times a week	IPC/nurses
Optimization of antimicrobial treatments	Pharmacist incorporation	Guide update	Guides compliance	Guides compliance	Quarterly	pharmacist/infectious diseases practitioner
Antimicrobial consumption	By drug per semester	By drug per semester	By hospital ward and drug monthly	By hospital ward and drug monthly	Semestral until 2109, monthly since 2020	pharmacist
Education	Face-to-face	Face-to-face	Virtual and face-to-face	Virtual and face-to-face	Each 15 days	IPC/ASP group
Feedback	x	x	x	X	Monthly	IPC

Quantification of the results of the interventions is done using the following indicators:

- Incidence density (ID) of infection by ERC: number of infections by ERC in patients hospitalized for more than 72 h per 1,000 patient days.
- ID of nosocomial colonization by ERC: number of colonization (nondoubled) by MRO in patients hospitalized for more than 72 h per 1,000 patient days.
- Incidence of community or other hospital colonization by ERC: number of colonizations (nondoubled) by MRO detected <72 h of admission per 1,000 discharges.
- ID of ventilator-associated pneumonia (VAP): number of VAP per 1,000 ventilator days.
- ID of central line-associated bloodstream infection (CLABSI): number of CLABSI per 1,000 central line days.
- ID of catheter-associated urinary tract infection (CAUTI): number of CAUTI per 1,000 catheter days.
- Hand hygiene compliance rate: number of appropriate hand hygiene episodes/total number of opportunities per 100.
- Hospital hygiene compliance rate: number of disappeared marks/total number of marks made per 100.
- Contact isolation compliance rate: number of contact isolations complied with/total number of isolation opportunities per 100.
- Bundle compliance rate: number of bundles complied with (discriminated by invasive device)/total number patients eligible for a bundle per 100.
- Antimicrobial consumption: Defined Daily Doses (DDD) per 100 patient days. The indicator is expressed as total antimicrobial consumption, and consumption of carbapenems, colistin, piperacillin-tazobactam, and third- and fourth-generation cephalosporins.
- Education: number of persons received education or capacitation.

## Statistical analysis

Categorical variables were presented as numbers and percentages. Continuous variables were presented as mean and standard deviation (SD) or median and interquartile range (IQR) according to data distribution.

To compare results of continuous variables, *t* test or Mann-Whitey test was performed, in line with type of distribution. Categorical variables results were analyzed using χ^2^ or Fisher’s exact test.

Numerators and denominators of infection episodes were registered using Epicontrol version 7, a computerized epidemiology and infection control surveillance record system.

Incidence rates were assessed with a Poisson regression model with patient days as the offset variable. Assumptions of the model were checked and satisfied. Pairwise comparisons by year and semester were evaluated with the Tukey method for multiple comparisons. Alpha was set at the 0.05 level.

Analysis was performed with R Studio v. 2023.03.1

## Results

**Figure 1.**
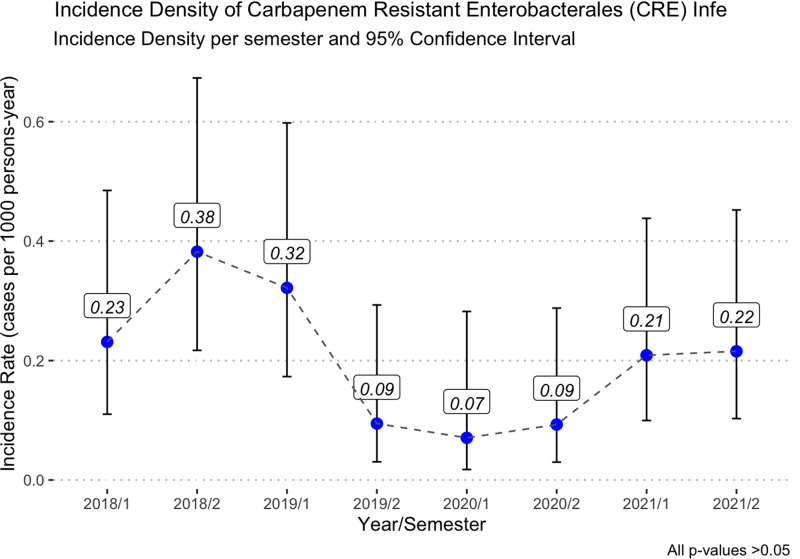
Incidence density of carbapenem-resistant Enterobacterales infection.

Synergy between an ICP and ASP achieved controlled rate of CRE infections finding the lowest levels during 2020 (0.08 episodes/1,000 patient days) (Figure 1). Colonization rate remained stable throughout the study period (Table 2).

**Table 2.** Results indicators

	1º semester 2018	2º semester 2018	1º semester 2019	2 semester 2019	1º semester 2020	2º semester 2020	1º semester 2021	2º semester 2021
Patient days (PD)	30281	31387	31082	31736	28341	32313	33511	32473
Hospital discharges (HD)	6442	6921	6441	6788	5577	6107	5845	6761
**CRE**								
Infection (*n*)	7	12	10	3	2	3	7	7
Infection Rate Ratio* (N°/ PD *1000)	0.23	0.38	0.32	0.09	0.07	0.09	0.21	0.21
Rate Ratio Colonization <72 hs after admission Nº/HD*1000 (nº)	0.93 (6)	1.01 (7)	0.93 (6)	0.29 (2)	0.18 (1)	0.33 (2)	0.68 (4)	0.59 (4)
Rate Ratio Colonization >72 hs after admission Nº/PD*1000 (nº)	0.72 (22)	0.60 (19)	0.70 (22)	0.28 (9)	0.18 (5)	0.25 (8)	0.65 (22)	0.18 (6)
**Device-associated Infections**								
VAP rate (‰)	5.27	8.85	3.19	3.03	2.70	9.22	4.93	3.94
CLABSI rate (‰)	1.36	1.52	1.83	1.74	2.41	3.40	1.89	1.48
CAUTI rate (‰)	2.64	1.23	1.82	2.39	1.76	1.96	2.69	3.36

CRE, Carbapenem-resistant Enterobacterales; VAP, ventilator-associated pneumonia; CLABSI, Catheter-associated bloodstream infection; CAUTI, Catheter-associated urinary tract infection.

There was a significant reduction in VAP rates between 2019 and 2021 (7.18 vs. 4.62 episodes per 1,000 ventilator days; *p* = 0.02). Notably, the rate had doubled in 2020, likely related to the COVID-19 pandemic (Table 2). There was no difference in CLABSI and a nonstatistical increase in the ID of CAUTI. Compliance with care bundles increased gradually during the study period achieving rates >85% by 2021.

**Figure 2.**
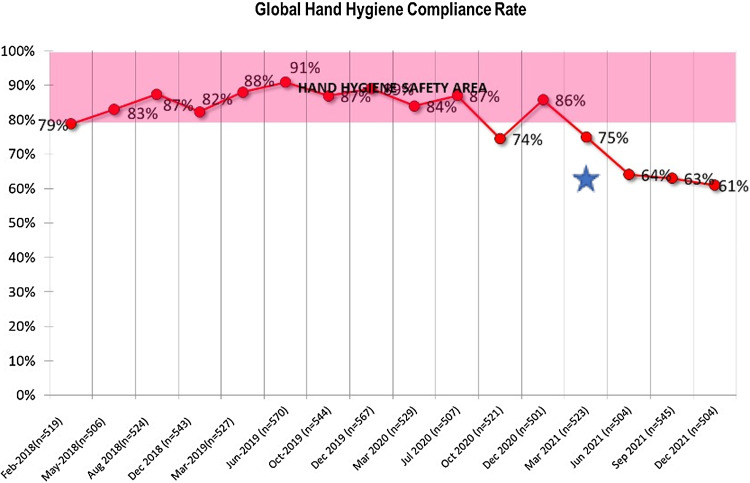
Hand hygiene compliance rate. *Note:* Adherence to hand hygiene moments and technique. The blue star marks the point in time when observations via cameras was added to direct observation.

Hand hygiene adherence rate was over 80% during 2018 to 2020 (Table 3). Thereafter, there was a reduction in the compliance rate, most likely linked to the modification in the observation method (Figure 2). Hospital hygiene adherence rate was consistently over 90%.

**Table 3.** Process indicators

	1 sem 2018	2 sem 2018	1 sem 2019	2 sem 2019	1 sem 2020	2 sem 2020	1 sem 2021	2 sem 2021
Hand hygiene compliance rate % (n)	81% (775/1224)	85% (854/1257)	91% (926/1021)	89% (1772/1985)	85% (881/1036)	83% (1699/2058)	79% (823/1043)	70% (731/1049)
Hospital hygiene compliance rate % (n)	89.5% (3095/3544)	90% (2837/3140)	93% (2881/3072)	87% (3756/4288)	92% (3461/3776)	92% (3963/4288)	92% (3780/4118)	90% (4540/5027)
Contact isolation compliance rate % (n)	64% (415/647)	87% (714/818)	69% (70/102)	NA	NA	NA	NA	NA
CC Bundle compliance rate % (n)	75% (181/241)	68% (185/272)	78% (185/236)	67% (558/827)	86% (231/267)	81% (461/568)	82% (392/478)	86% (253/293)
MV Bundle compliance rate % (n)	69% (45/65)	77% (64/83)	89% (55/62)	85% (199/233)	94% (87/92)	60% (167/275)	84% (222/265)	95% (109/115)
UC Bundle compliance rate (%)	60% (54/90)	84% (123/146)	88% (100/113)	88% (357/407)	91% (113/124)	87% (280/320)	86% (267/310)	85% (122/143)
Global antimicrobial consumption	1877.9	1931.37	1967.13	1897.09	2007.53	2434.21	2382.02	1960.74
Carbapenem consumption	281.53	356.12	349.73	286.62	359.49	345.94	305.48	332.99
Colistin consumption	108.74	142.84	120.04	116.59	123.16	181.52	274.53	138.58
Piperacillin-tazobactam consumption	399.98	399.05	377.76	392.10	286.77	400.95	455.37	351.19
3° + 4° generation cephalosporin consumption	278.73	303.35	270.73	346.86	413.15	537.74	444.33	358.46
Education and training (nº person)	138	531	341	321	221	202	204	216

Antimicrobial consumption in DDD/100 PD.

Compliance with contact isolation was 76% in 2018 and 69% in 2019. Given the extension of isolation precautions during the COVID-19 pandemic, data from 2020 and 2021 were not included (Table 2).

Antimicrobial consumption increased slightly during the study period (15%). Among high-impact antimicrobials, however, only colistin consumption increased (Table 3).

The number of persons who received education and training in prevention and infection control was stable during the study period. We included computerized tools on topics such as hand hygiene (moments and technique) and isolation precautions, which were mandatory for the entire hospital staff and allowed the continuity of training despite the pandemic.

## Discussion

To our knowledge, we are the first to show the results of 4 years ASP and PCI working jointly to fight AMR in Argentina.

Our study shows that it is possible to achieve a stable CRE infection rate by implementing our strategy despite Argentina´s situation—high CRE incidence—and COVID-19 pandemic.

We believe that these results were the consequence of some differential components of the programs which are seldom found together in our country: first, management support was crucial in the priorization of these programs in means of funds designation and unique coordination; second, screening CRE on admission and in high-risk areas with prompt identification and isolation’ third, inclusion of a clinical pharmacist in 2018, allowing us to quantify the ASP actions, finally, encouraging interdisciplinary work groups with healthcare personnel to audit and maintain adherence to hand hygiene, hospital hygiene, and bundles for the prevention of device-associated infections and the use of antimicrobials in order to establish continuous improvement strategies over time.

National rates of HAI are higher than those reported in our study (VAP 11,28 infections/1,000 ventilator days; CLABSI 5,57 infections /1,000 central line days), highlighting the importance of allocating resources and prioritizing IPC and ASP programs in healthcare facilities.

During the peak of COVID-19 pandemic, as in most institutions,^19–22^ HAI rates increased in our facility. However, having a working and robust ICP program allowed us to control the situation and even reduce the rates of VAP and CLABSI during the study period.

Hand and hospital hygiene compliance rates were consistently high. We attribute this outcome to the commitment of our staff, their effective teamwork, and the cultivation of a safety-centric culture. We further enhanced monitoring measures by adding an external audit for hospital hygiene and video cameras for hand hygiene. This strategy was included in new campaigns, training, and education. Continuous training of hospital hygiene staff together with audit and feedback have proved to be key factors in the continued success of this standard. At the very beginning, it was hard to convince staff of the importance of both programs. Today, the main issue is to sustain already implemented measures.

Global antimicrobial consumption remained stable, despite certain variation among antimicrobial families. Argentina regretfully lacks hospital comparators, and antimicrobial consumption is reported to be between 11 and 36 DDD per 1,000 inhabitants per day.^23^

ANLIS Malbrán—Argentina´s Institute of Reference for antimicrobial resistance—reported 32.4% of carbapenem-resistant *K. pneumoniae* from 43.495 nosocomial isolates.^12^ Such situation urges the need to restrict unnecessary antimicrobial use and to optimize infection prevention and control measures.^13^

A recent publication determined that the multi-resistance load could be reduced by 85% with the implementation of an ICP that emphasizes hand hygiene and hospital hygiene, with strong support from an ASP.^4^ Such a strategy is cost-effective and not resource-prohibitive. This evidence is reinforced by a metanalysis,^14^ which showed that ASP, when implemented alongside infection control measures, are more effective than implementation of ASP alone on reduction of AMR. Studies co-implementing a hand hygiene program with an ASP have reported a reduction of 66% in antibiotic resistance versus 17% in studies without co-implementation of hand hygiene interventions, supporting the so-called “butterfly effect” of hand hygiene.^14^

As established by Dik and colleagues,^15^ controlling the prevalence of AMR infections can only be achieved through a holistic approach exemplified by the tripartite model of antimicrobial stewardship, infection prevention, and diagnostic stewardship.

Although Argentina is among the 54% of countries with a national IPC program,^4^ only selected institutions have robust systems in place to implement and disseminate IPC practices. Even where IPC and ASP programs are in place, they are often not able to function appropriately and sustainably in an enabling environment. Additionally, they often function independently and not synergistically. Moreover, particularly in LMICs, facilities frequently lack full-time IPC professionals, an allocated IPC budget, routine microbiological laboratory support, and appropriate workload, staffing, and bed occupancy.^10^

It is common knowledge that colonization by MRO increases the risk of subsequent infection by these organisms.^16–18^ Thus, colonized patients comprise a high-risk group for infection by MRO, as well as an important source for transmission of MRO in the hospital setting. Surveillance of MRO microorganisms constitutes an opportunity to break the chain of transmission^18^. The application of containment strategies for colonized patients and verification of compliance likely bolstered the success of CRE control.

Our study has limitations. First, it was carried out in a single institution, which has an established safety culture. Hence, the results might not be applicable to other healthcare institutions. However, we are certain that our results demonstrate that coordinated actions of both programs have a favorable impact on AMR. Second, the COVID-19 pandemic may have acted as a confounder, altering the course of the study variables. In the pandemic setting, synergy between both programs allowed us to easily return to prepandemic rates of HAI.

We did not analyze individual factors in relation to the results. However, we strongly believe that attaining high levels of compliance with process indicators such as hand hygiene, hospital hygiene, isolation precautions, and care bundles in articulation with antimicrobial stewardship measures was critical in CRE control.

## Conclusions

Our study suggests that achieving a reasonable CRE infection rate in LMICs hinges on the sustained and beneficial impact of both IPC and AS programs when implemented collaboratively by an interdisciplinary team. Success would have been unlikely without likely our senior leadership’s commitment to both critical programs. Further studies in LATAM are needed to support our results and demonstrate the importance of a synergistic approach in controlling CRE.

## Supporting information

Cornistein et al. supplementary material Cornistein et al. supplementary material

## Supplementary material

For supplementary material accompanying this paper visit http://doi.org/10.1017/ash.2023.439. click here to view supplementary material
