# Supplementary material for: Synergy between infection control and antimicrobial stewardship programs to control carbapenem-resistant Enterobacterales
Source: Antimicrob Steward Healthc Epidemiol. 2023 Sep 26;3(1):e162. doi: 10.1017/ash.2023.439 (PMC10523542; doi:10.1017/ash.2023.439)
Supplement: Cornistein et al. supplementary material [file S2732494X23004394sup001.docx]

Appendix 1. VIHDA Program considers the following criteria

**Ventilator associated pneumonia (VAP)**

1. At least one of the following signs and symptoms:

- Impaired gas exchange (expressed as Pa/Fi deterioration or need to increase PEEP or FiO2 after 48 hours of stability)

- Fever (>38ºC) with no other recognized cause

–Leukopenia (<4000) or leukocytosis (>12,000)

2. Presence of new or progressive pulmonary infiltrate on persistent chest radiograph more than 48 hours

3. purulent tracheal aspirate (TA)

4. Positive microbiological technique (tracheal aspirate >= 105 CFU of a potentially pathogenic microorganism, or bronchoalveolar lavage > 104 CFU, or protected brushing > 103).

**Catheter associated urinary tract infection (CAUTI)**

They must meet the following 3 points:

1. Patient with urinary catheter for > 48 consecutive hours on the day of the event and also:

a) who was present for any part of the day of the event

b) had been removed the previous day.

2. At least one of the following signs or symptoms with no other recognized cause: fever (>38ºC), suprapubic pain, pain or tenderness of the costovertebral angle, urgency to micturition, dysuria and/or increased urinary frequency

3. Positive urine culture with <105 CFU/ml with no more than 2 species of microorganisms

**Catheter associated bloodstream infection (CLABSI)**

A central venous access-associated bacteremia (BACT-CC) occurs in a patient who meets any of the following criteria:

1. Clinical signs (fever, hypotension, pain, erythema, discharge, increased temperature) with positive culture of the catheter tip by semiquantitative technique of Maki ≥ 15 CFU and/or quantitative of Brun Buisson , plus positive blood cultures from samples taken before removing the catheter with a count ≥1000, or positive yeasts.

2. Positive peripheral blood culture plus catheter tip culture by Maki technique ≥ 15 CFU, in the absence of another focus responsible for bacteraemia.

3. Positive blood culture plus clinical signs of sepsis plus imaging findings (US Doppler , CT, MRI, phlebography) of central vein thrombosis or collection compatible with septic embolus or fungoma related to the presence of central catheters.

4. Positive quantitative retroculture with a differential colony count ≥5 to 10 times (or positivity time > 2 hours) in relation to the colony count of the same peripheral blood culture germ associated with a clinical picture.

5. Positive blood culture plus positive culture from puncture aspiration of the catheter insertion area.
